# Protective effects of Salubrinal against H_2_O_2_-induced muscle wasting via eIF2α/ATF4 signaling pathway

**DOI:** 10.3389/fphar.2025.1607606

**Published:** 2025-06-27

**Authors:** Siming Lin, Jingying Wu, Guili Lian, Weibin Wu, Weixiao Chen, Ai Chen, Li Luo, Liangdi Xie

**Affiliations:** ^1^ Department of Emergency, The First Affiliated Hospital, Fujian Medical University, Fuzhou, China; ^2^ Department of Emergency, National Regional Medical Center, Binhai Campus of The First Affiliated Hospital, Fujian Medical University, Fuzhou, China; ^3^ Fujian Hypertension Research Institute, The First Affiliated Hospital of Fujian Medical University, Fuzhou, China; ^4^ Clinical Research Center for Geriatric Hypertension Disease of Fujian Province, The First Affiliated Hospital of Fujian Medical University, Fuzhou, China; ^5^ Department of Geriatrics, The First Affiliated Hospital of Fujian Medical University, Fuzhou, China; ^6^ Branch of National Clinical Research Center for Aging and Medicine, The First Affiliated Hospital of Fujian Medical University, Fuzhou, China; ^7^ Department of Geriatrics, National Regional Medical Center, Binhai Campus of the First Affliated Hospital, Fujian Medical University, Fuzhou, China

**Keywords:** sarcopenia, aging, skeletal muscle atrophy, endoplasmic reticulum stress, salubrinal

## Abstract

**Background:**

Endoplasmic reticulum stress (ERS) plays a critical role in skeletal muscle physiology and pathology, though the precise mechanisms remain unclear. Salubrinal, a selective inhibitor of eIF2α dephosphorylation, has been shown as a potential therapeutic agent for various conditions, but its effects on sarcopenia are not well understood. This study investigated the protective effects of salubrinal against H_2_O_2_-induced muscle cell injury and its impact on the eIF2α/ATF4 signaling pathway.

**Methods:**

Gastrocnemius muscle samples from aged mice were used and cultured C2C12 myotubes were also used to explore the effects of Salubrinal through Western blotting, immunofluorescence, and apoptosis assays.

**Results:**

Our results demonstrated that H_2_O_2_ treatment induced significant muscle cell damage, evidenced by reduced MHC1 expression and increased apoptosis. Salubrinal, in a concentration-dependent manner, mitigated these effects, preserving MHC1 expression and reducing apoptosis. Furthermore, salubrinal enhanced the expression of p-eIF2α and ATF4, suggesting that its protective effects are mediated through the eIF2α/ATF4 pathway.

**Conclusion:**

These findings highlight salubrinal’s potential as a therapeutic agent for muscle wasting conditions, particularly those related to oxidative stress and ERS.

## 1 Introduction

Sarcopenia, a condition linked to aging, involves a gradual and widespread decline in muscle mass and strength or function (Dent et al., 2018). An aging global population poses a major public health challenge, resulting in a rise in age-related illnesses, a decline in life quality, and considerable economic strain from escalating healthcare expenses and the demand for long-term care. Therefore, the prevention and treatment of sarcopenia are very important public health issues, and much attention should be paid to (Cruz-Jentoft and Sayer, 2019).

Current preventive and curative measures for sarcopenia include targeted exercise therapy (Pahor et al., 2014; Bann et al., 2016), adequate nutritional interventions (Tieland et al., 2012; Cermak et al., 2013), and pharmacological treatments. Exercise, in particular, is widely recognized as one of the most effective interventions for sarcopenia, as it helps maintain muscle mass, strength, and function through resistance and aerobic training. The development of sarcopenia has long been a focus of scientific study due to the incomplete understanding of the molecular processes involved, and the current absence of successful treatments for preventing or treating sarcopenia. The pathophysiological mechanisms of sarcopenia in the elderly are complex. These mechanisms include oxidative stress, mitochondrial dysfunction, imbalance of protein metabolism, inflammation, apoptosis, satellite cell dysfunction, decreased motility, diminished neuromuscular function, and aging-related humoral changes (Li et al., 2018). The complex pathophysiological mechanisms that can occur during human aging provide a wide range of potential therapeutic targets for sarcopenia treatment (Cruz-Jentoft and Sayer, 2019); however, the discovery of multiple targets and signaling pathways in the pathogenesis of sarcopenia has also complicated the clinical transformation. Hence, it is of utmost importance to recognize the junction of these processes.

Sarcopenia is an involuntary loss of skeletal muscle mass and strength associated with aging (Shaw et al., 2017). This condition results in a decline in bodily functions and reduces the stability and stress capacity of the internal environment, leading to senescence. ER stress plays a significant role in the aging process, not only through the accumulation of damaged proteins (Matos et al., 2015) but also via other mechanisms, such as the involvement of the inner nuclear membrane protein SUN2, which activates ER stress in premature aging (Vidak et al., 2023). Additionally, aging-related mitochondrial dysfunction and sphingolipid imbalances have been shown to exacerbate ER stress, further contributing to cellular decline (Chen et al., 2023). Ikeyama et al. (Ikeyama et al., 2003) reported an increased basal expression of C/EBP homologous protein (CHOP) in the liver and pancreas of mice during senescence. These results suggest that chronic ERS is common in senile bodies.

CHOP is a well-characterized transcription factor upregulated during severe and sustained ER stress, primarily through the PERK-eIF2α-ATF4 pathway (Ikebe et al., 2020). Elevated CHOP expression promotes apoptosis by suppressing anti-apoptotic proteins (e.g., Bcl-2), inducing pro-apoptotic factors (e.g., Bim), and increasing oxidative stress through reactive oxygen species production (Ghosh et al., 2012; Noh et al., 2012). Thus, CHOP plays a pivotal role in the cellular injury associated with prolonged ER stress, including skeletal muscle pathology.

In eukaryotic cells, the endoplasmic reticulum plays a vital role as a membrane organelle in the processes of protein synthesis, folding, and secretion. Either endogenous or exogenous stimuli leads to dysfunction of the endoplasmic reticulum in folding proteins. Numerous improperly folded proteins build up in the endoplasmic reticulum, causing a disruption in its balance, known as ERS (Lam et al., 2020). The activation of the unfolded protein response (UPR) controls the subsequent pathway through three primary pathways: protein kinase RNA-like ER kinase (PERK)-eIF2α, inositol-requiring enzyme 1α (IRE1α)-x-box-binding protein 1 (XBP-1), and activating transcription factor 6 (ATF6) (Kato and Nishitoh, 2015). In turn, it regulates apoptosis, inflammation, autophagy, oxidative stress, etc., and helps stressed cells restore homeostasis (Chen and Cubillos-Ruiz, 2021). In the PERK-eIF2α pathway, ATF4 functions as a critical downstream effector. Upon eIF2α phosphorylation, the translation of ATF4 is selectively upregulated, promoting the expression of genes involved in apoptosis, autophagy, and oxidative stress response (Rzymski et al., 2009; Hart et al., 2012). This highlights ATF4’s central role in restoring cellular homeostasis under ER stress conditions.

Salubrinal is a synthetic and highly selective small molecule inhibitor of eIF2α dephosphorylation (480 Da, C21H17Cl3N4OS), which can activate intracellular eIF2α phosphorylation and thus reduce ERS-induced apoptosis. Studies have demonstrated that salubrinal has therapeutic effects on tumors (Kardos et al., 2020; Alsterda et al., 2021), arthritis (Wang et al., 2021), paraquat poisoning (Wang et al., 2018), and neuroprotective effects in cardiopulmonary resuscitation models and cerebral hemorrhage models (Masuri et al., 2020; Zhang et al., 2020). Although studies have confirmed that salubrinal can alleviate many diseases by modulating the eIF2α-related signaling pathway, its role in senile sarcopenia is unclear.

Although it is known that ERS plays a significant role in muscle wasting, precisely regulating ERS to protect muscle cells from damage, remains an unresolved issue. Furthermore, the specific role of the eIF2α/ATF4 signaling pathway, which is part of the ERS response, in muscle wasting is not yet clear. Thus, exploring potential drugs that can effectively modulate this pathway to alleviate muscle cell damage is crucial for developing new therapeutic strategies.

This study aims to explore how salubrinal can protect muscle cells from injury caused by H_2_O_2_ and to investigate if this protection is linked to the eIF2α/ATF4 signaling pathway. This study aims to uncover the molecular mechanisms through which salubrinal reduces muscle cell damage by examining its effects on MHC1, p-eIF2α, ATF4 expression, and cell apoptosis. We anticipate that salubrinal will effectively inhibit H_2_O_2_-induced muscle cell injury, reduce cell apoptosis, and exert its protective effects by modulating the eIF2α/ATF4 signaling pathway. This finding will not only offer a new perspective on the molecular mechanisms of muscle wasting but also provide scientific evidence for the development of novel therapeutic drugs targeting muscle wasting.

## 2 Materials and methods

### 2.1 Antibodies and reagents

Immunoblotting, immunohistochemistry, and immunofluorescence were performed using the following primary antibodies:Cell Signaling Technology in MA, United States provided the anti-eIF2α antibody (D7D3), anti-Phospho-eIF2α antibody (Ser51) (D9G8), anti-CHOP antibody (L63F7), and anti-beta-actin antibody (#3700). Proteintech in Wuhan, China supplied the anti-GAPDH antibody (60004-1-Ig) and anti-beta-Tubulin antibody (66240-1-Ig). The anti-MHC1 antibody was acquired from the Developmental Studies Hybridoma Bank (DSHB) in Iowa, United States. Anti-Atrogin-1 antibody (ab168372) and anti-GRP78 antibody (ab21685) were purchased from Abcam (MA, United States). Proteintech (Wuhan, China) provided the Anti-MuRF-1 antibody (55456-1-AP) and the anti-ATF4 antibody (60035-1-Ig). The secondary antibodies labeled with Alexa Fluor 594 (ZF-0516) and Alexa Fluor 488 (ZF-051) were bought from Zhongshan Golden Bridge in Beijing, China. The DeadEnd™ Fluorometric TUNEL System (G3250) was acquired from Promega in Wisconsin, United States. The Hoechst 33342/PI dual staining kit was acquired from Solarbio in Beijing, China. Salubrinal was purchased from MedChemExpress (shanghai, China). The hydrogen peroxide solution was bought from Sigma-Aldrich in Missouri, United States.

### 2.2 Animal model and experimental design

Previous work by our team has established a biospecimen library of gastrocnemius derived from elderly Sarcopenia mice, including 10 5-month-old young mice, 9 15-month-old middle-aged mice and 14 22-month-old elderly mice (Wu et al., 2023). All animal experiments were conducted using male C57BL/6J mice. For the purpose of this research, 8 mice from each of the 3 groups were randomly chosen to have gastrocnemius muscle samples taken for further testing. Approval from the Animal Ethics Committee was obtained for all experiments (Ethics No. FJMU IACUC 2021-0385).

### 2.3 Immunohistochemistry staining

Tissues were processed by dehydration, paraffin embedding, and sectioning into 10 μm slices for immunohistochemistry to assess CHOP expression. Although 10 μm sections were used in this study based on our previous experiments, we acknowledge that thinner sections (3–5 μm) may provide better resolution for screening protein expression. Future studies will implement thinner sections to improve the visualization and accuracy of protein expression. We performed immunohistochemistry as previously described (Wu et al., 2023). Briefly, after dewaxing and rehydrating, sections were treated with 3% hydrogen peroxide, blocked with 10% bovine serum albumin, and incubated with CHOP Mouse mAb (1:1000) at 4°C overnight. Following incubation with secondary antibodies, sections were developed with DAB, counterstained with hematoxylin, and examined under a Nikon Eclipse E200 microscope, with Image-Pro Plus software for image analysis. CHOP expression was evaluated based on a semi-quantitative method considering staining intensity and the percentage of positive cells.

### 2.4 Cell culture, differentiation and treatment

Procell Life Science and Technology Co., Ltd. supplied the Mouse C2C12 myoblast cell line. Cell culture and differentiation were performed as described previously (Wu et al., 2023). The cells were cultured in high-glucose dulbecco’s modified eagle medium (DMEM) with 10% fetal bovine serum (FBS) and 1% penicillin-streptomycin at 37°C with 5% CO_2_ until they reached over 90% confluence. To induce the formation of myotubes, the medium was replaced with DMEM containing 2% horse serum and was refreshed every day for a period of 5 days. Immunofluorescence identified differentiated myotubes, which were then subjected to various treatments based on experimental conditions. Subsequently, they were subjected to a 4-h period of starvation in serum-free medium before any intervention. Salubrinal was introduced 1 h prior to the intervention, followed by the addition of H_2_O_2_. Furthermore, the serum-free medium containing H_2_O_2_ or distilled water (ddH_2_O) or dimethyl sulfoxide (DMSO) or salubrinal or salubrinal + H_2_O_2_ was modified on a daily basis throughout the experiments using a high sugar medium (Siu et al., 2009). Then experiments assessed the effects of H_2_O_2_ concentration, H_2_O_2_ exposure duration, and combinations of H_2_O_2_ and salubrinal on myotube stimulation over specific time frames.

### 2.5 Immunofluorescence staining

Sterile coverslips were used to culture myoblasts, which were then induced to undergo differentiation into myotubes. Immunofluorescence staining was performed based on the technique we used and described previously (Yu et al., 2012; Wu et al., 2023).

In short, myoblasts were placed on clean coverslips and prompted to transform into myotubes, which were later treated with 4% paraformaldehyde for 15 min at room temperature, followed by permeabilization using 0.25% TritonX-100 for 10 min at 4°C, and blocking with 5% bovine serum albumin (BSA) for 30 min at room temperature. Following this, myotubes were left to incubate at 4°C overnight with a 1:100 dilution of an anti-MHC1 antibody. After the initial antibody treatment, the myotubes were rinsed with phosphate buffer saline (PBS) and exposed to a secondary antibody tagged with Alexa Fluor 488 (at a 1:200 dilution) for 1 hour. Subsequently, they were stained with 4′,6-diamidino-2-phenylindole (DAPI) (provided by Cell Signaling Technology, United States) at a concentration of 1 μg/mL. Images with fluorescence were captured using the Nikon Eclipse TS2R microscope from Nikon in Japan. For immunofluorescence, quantitative analysis was performed using ImageJ software. Fluorescence intensity was measured across at least four randomly selected fields per sample, and the number of positive cells was counted. The mean fluorescence intensity and percentage of positive cells were calculated to evaluate the expression of the target proteins.

### 2.6 Western blot

Western blot analysis was conducted in accordance with the previously described procedure (Chen et al., 2009; Zhuang et al., 2019). The process is briefly described as follows: extract proteins, denature them in boiling water, and detect the expression of target proteins through the steps of gel preparation, sample loading, electrophoresis, membrane transfer, closure, incubation of antibodies, membrane washing, development and fixation. The antibodies for eIF2, p-eIF2α, CHOP, Atrogin-1, glucose-regulated protein 78 (GRP78), MuRF-1, and ATF4 were diluted at a rate of 1:1000, while the antibodies for MHC1, GAPDH, β-Tubulin, and β-actin were diluted at a rate of 1:5000. Explanation of Protein Expressions Evaluated: eIF2α and p-eIF2α: To assess the involvement of the ER stress response, as salubrinal is known to modulate the phosphorylation of eIF2α. CHOP: To measure apoptotic signaling induced by prolonged ER stress. Atrogin-1 and MuRF-1: Key markers of muscle atrophy, their upregulation indicates muscle protein degradation. GRP78: A hallmark of ER stress, GRP78 levels provide insights into the overall activation of ERS. ATF4: As a downstream effector of the eIF2α pathway, ATF4 plays a crucial role in stress responses, including oxidative stress and apoptosis. MHC1: To evaluate muscle cell differentiation and the impact of H_2_O_2_ on myotube integrity. GAPDH, β-Tubulin, and β-actin: These were used as internal controls to ensure consistent protein loading across samples.

### 2.7 Hoechst 33342/PI double staining assay

Myoblasts were placed on plates with 6 or 12 wells, stimulated to undergo differentiation into myotubes, and subjected to the prescribed treatment. The Hoechst 33342/PI double stain kit was used to measure cell death in accordance with the manufacturer’s instructions. Each well’s medium was substituted with 1 mL of staining buffer comprising 5 μL of Hoechst 33342 and 5 μL of PI, subsequently subjected to a 30-min incubation at 4°C without any illumination. Subsequently, the cells were rinsed with PBS and examined under the Nikon Eclipse TS2R microscope (Nikon, Japan). The ImageJ software was used to randomly select four yields per well for counting the stained cells.

### 2.8 TUNEL analysis

Plates with 6 or 12 wells were utilized to position myoblasts, which were then stimulated to undergo differentiation into myotubes and subjected to the prescribed treatment. The DeadEnd™ Fluorometric TUNEL (Promega, United States) was utilized to conduct the TUNEL assay, following the instructions provided in its manual. The images were captured under a microscope to observe the green fluorescence emitted by TUNEL-positive cells and the blue fluorescence emitted by the total DNA. Using ImageJ software, four fields per well were randomly chosen to count stained cells. The percentage of apoptotic cells was calculated using the following formula: (Number of TUNEL-positive cells/Total number of cells) × 100%.

### 2.9 Statistical analysis

The experimental data were established using Excel and all data were statistically analyzed using the SPSS 13.0 software system. Measurements were expressed in mean standard deviation and differences were tested using one-way ANOVA and then pairwise comparisons with SNK-*q* test. The difference was statistically significant at *P* < 0.05.

## 3 Results

### 3.1 Expression levels of GRP78 and CHOP in gastrocnemius muscle of aged mice

**FIGURE 1:**
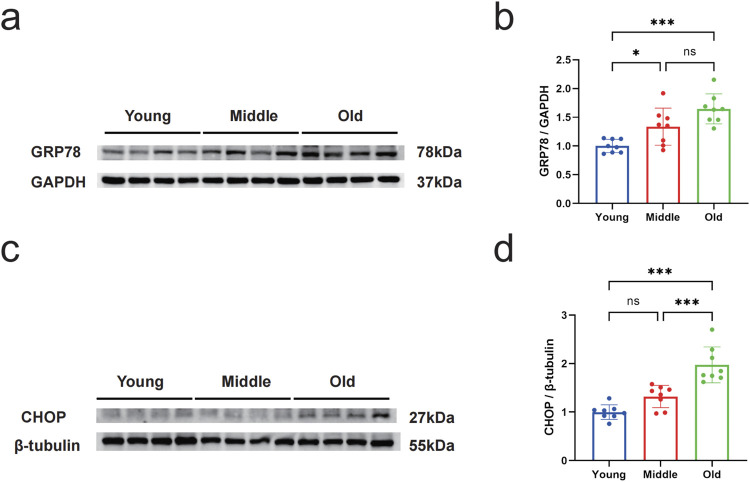
Changes in the expression levels of GRP78 and CHOP in the gastrocnemius muscle of mice during aging. **(a)** Western blot detection of GRP78 expression in gastrocnemius muscle of young, middle-aged, and old mice. GAPDH was used as the loading control. **(b)** Relative fold change in protein expression of GRP78 in the gastrocnemius muscle of young, middle-aged, and old mice. **(c)** Western blot detection of CHOP expression in gastrocnemius muscle of young, middle-aged, and old mice. β-tubulin was used as the loading control. **(d)** Relative fold change in protein expression of CHOP in the gastrocnemius muscle of young, middle-aged, and old mice. Data are presented as mean ± standard deviation. NS, no significance, *P < 0.05, **P < 0.01, ***P < 0.001, n = 8 mice per group.

**FIGURE 2:**
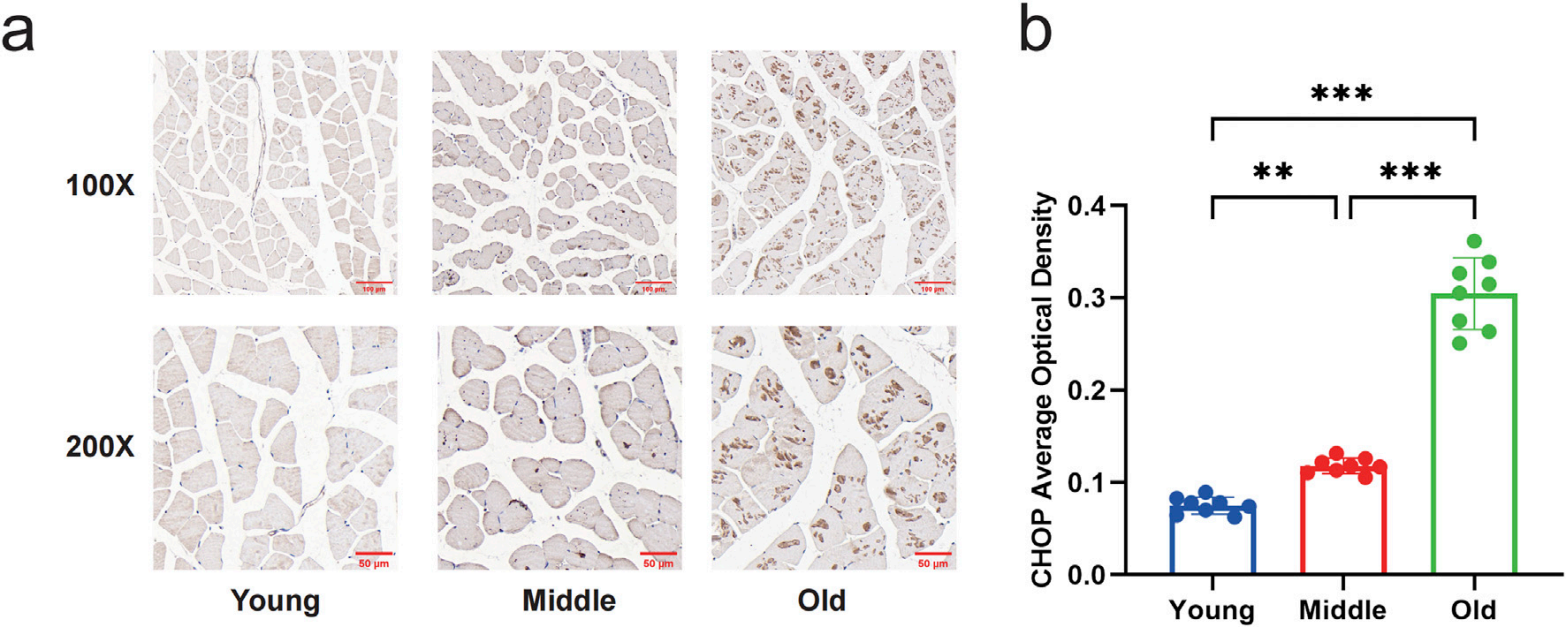
Changes in expression of CHOP in the gastrocnemius muscle of mice by immunohistochemical staining. **(a)** Immunohistochemical staining to compare the expression of CHOP in the gastrocnemius muscle of young, middle-aged, and aged mice (Magnification: ×100, ×200). There were no brownish-yellow granules in the gastrocnemius muscle of the young group; a few brown-yellow granules were seen in the gastrocnemius muscle of the middle-aged group; and a large number of brown-yellow granules were seen in the old group. **(b)** Quantitative comparison of the average optical density of CHOP in young, middle-aged, and old mice. Data are presented as mean ± standard deviation. ns, no significance, *P < 0.05, **P < 0.01, ***P < 0.001, n = 8 mice per group.

To measure the level of ERS, we examined the protein expression of GRP78 and CHOP by Western blot. The results indicated a significant increase in GRP78 protein levels in the gastrocnemius muscle of mice in the middle-aged and old-aged groups compared to the young group (*P* < 0.05) (Figures 1a,b). In the old-aged group, the protein level of CHOP in the gastrocnemius muscle of mice was significantly higher than in the young and middle-aged groups (*P* < 0.05) (Figures 1c,d). Immunohistochemical results of CHOP showed that no brownish-yellow granular deposits in the young group; a small amount of brownish-yellow granular deposits in the middle-aged group; and a large amount of brownish-yellow granular deposits in the aged group (Figure 2a). Histochemical grayscale (optical density (OD)) analysis showed that the level of CHOP protein in the middle-aged group was significantly increased (*P* < 0.05) compared with that of the young group. Further increased (*P* < 0.05) in the aged group (Figure 2b). Therefore, the above results suggest that ERS levels in the gastrocnemius muscle of mice gradually increased with increasing age.

### 3.2 Myotube cell identification

**FIGURE 3:**
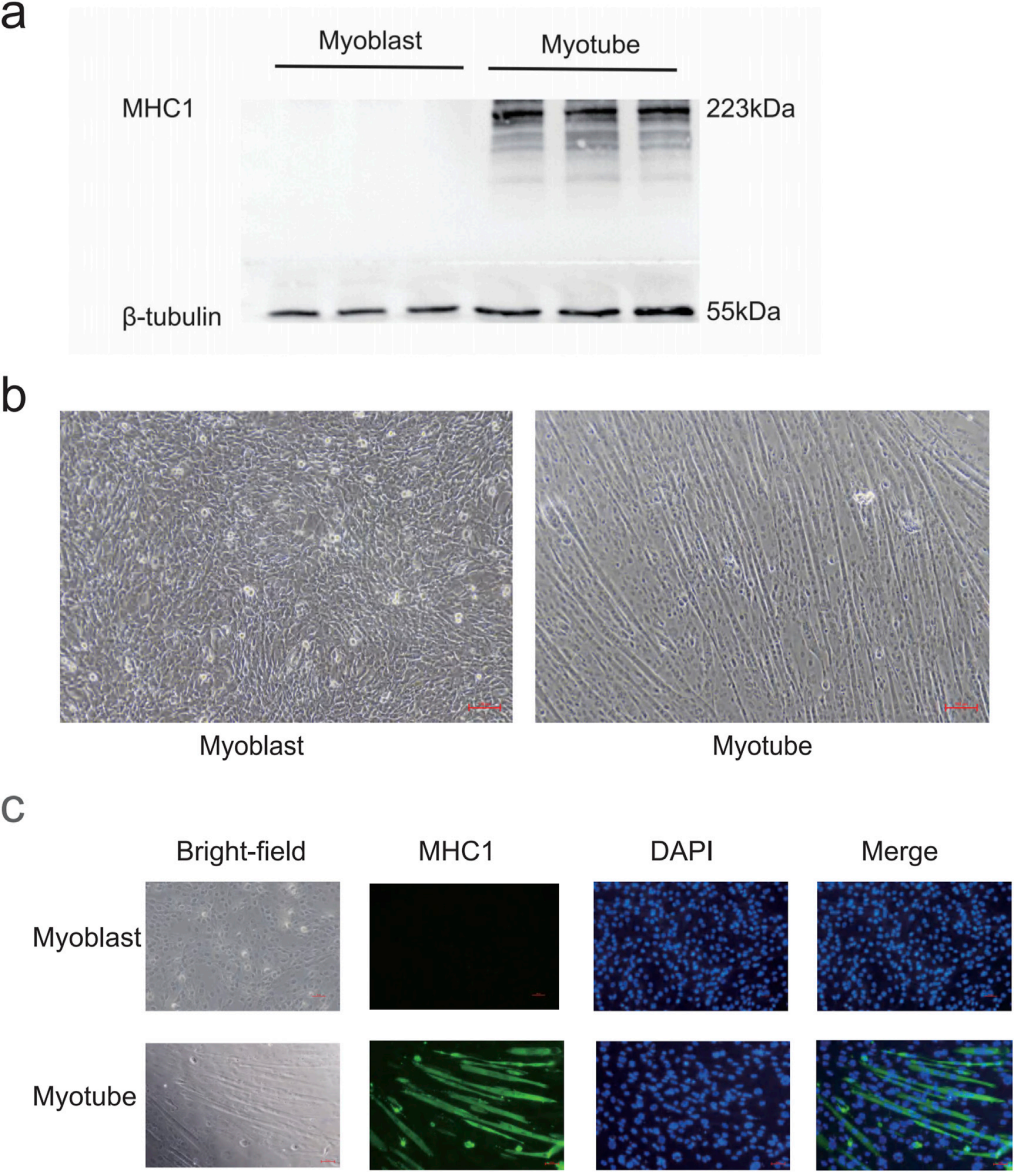
Myotube identification. **(a)** Western blot detection of MHC1 expression levels in C2C12 cells before and after differentiation. β-tubulin was used as the loading control. **(b)** Morphological changes of C2C12 cells before and after differentiation. Before differentiation, the cells are spindle-shaped, mononuclear, and have prominent nucleoli. After differentiation, the cells are in a fusion state, and the cells are oriented and arranged, neatly bundled, and multinucleated myotubes are formed. Scale bar = 100 μm (magnification ×100). **(c)** Immunofluorescence detection of MHC1 expression levels in C2C12 cells before and after differentiation. After differentiation, the cytoplasm of myotubes has green fluorescence (MHC1 positive), and blue is the nucleus (DAPI staining).

After 5 days of induced differentiation, C2C12 myoblasts could differentiate into mature skeletal muscle multinucleated myotubes. Under the microscope, the cells before differentiation were pike-shaped, mononuclear, with obvious nucleoli; after differentiation, the cells were fused, and the cells were oriented and arranged in bundles with multinucleated myotubes (Figure 3b); Western blot showed that myotubular cells specifically expressed the protein MHC1 before and after differentiation from absence to presence (Figure 3a); immunofluorescence shows the expression of specific protein MHC1 in the cytoplasm of myotube cells (Figure 3c). Based on the above results, myotubular cells were successfully differentiated.

### 3.3 H_2_O_2_ induces myotubular cell injury

**FIGURE 4:**
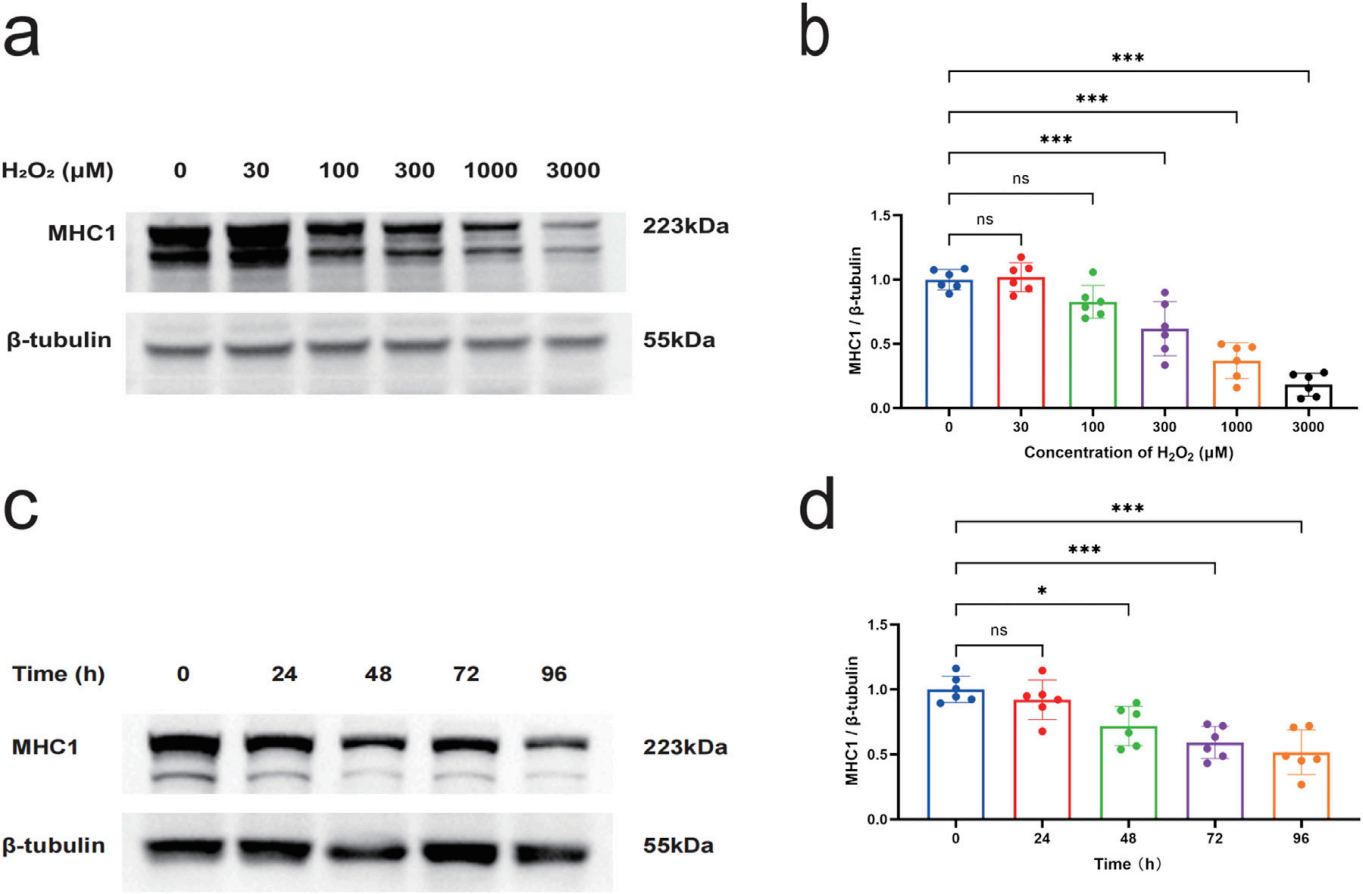
The effect of H_2_O_2_ on the expression level of MHC1 in myotube cells. **(a)** Western blot to detect the effect of different concentrations (0, 100, 300, 1000, and 3000 μmol/L) of H_2_O_2_ on the expression of MHC1 in C2C12 myotube cells for 48 h. β-tubulin was used as the loading control. **(b)** Quantitative analysis of the effect of different concentrations of H_2_O_2_ on the expression of MHC1 in C2C12 myotube cells for 48 h. **(c)** Western blot to detect the effect of 300 μmol/L H_2_O_2_ on the expression of MHC1 in C2C12 myotube cells at different times (0, 24, 48, 72, and 96 h). β-tubulin was used as the loading control. **(d)** Quantitative analysis of the effect of 300 μmol/L H_2_O_2_ on the expression of MHC1 in C2C12 myotube cells at different times. Data are presented as mean ± standard deviation. ns, no significance, *P < 0.05, **P < 0.01, ***P < 0.001, n = 6 independent experiments.

H_2_O_2_ is known as a non-radical reactive oxygen species that diffuses across cell membranes and increases intracellular levels of reactive oxygen species (Mougeolle et al., 2015), and H_2_O_2_ has been used to stimulate cells as a model to study cellular damage by oxidative stress (Barbieri and Sestili, 2012). In order to verify whether oxidative stress induces myotube injury *in vitro*, in this study, myotube cells were treated with H_2_O_2_, and myotube-specific expression of MHC1 was detected by Western blot to determine the injury of myotube cells. The results of Western blot showed that the stimulation of myotubes with H_2_O_2_ ranging from 30 to 3000 μmol/L for 48 h inhibited the expression of MHC1 protein significantly (*P* < 0.05) compared to the control group. The expression level of MHC1 was significantly reduced when the concentration of H_2_O_2_ reached 300 μmol/L and above, compared with the blank control group (*P* < 0.05) (Figures 4a,b). Stimulation of myotubes with a concentration of 300 μmol/L H_2_O_2_ for different times ranging from 24 to 96 h, it was found that H_2_O_2_ significantly reduced the expression of MHC1 in myotubes in a time-dependent manner, and the expression of MHC1 was significantly reduced when the intervention time reached 48 h and above (*P* < 0.05) (Figures 4c,d). Therefore, we chose 300 μmol/L H_2_O_2_ treatment for 48 h as the subsequent experimental condition.

### 3.4 Effect of Salubrinal on H_2_O_2_-induced myotube damage

**FIGURE 5:**
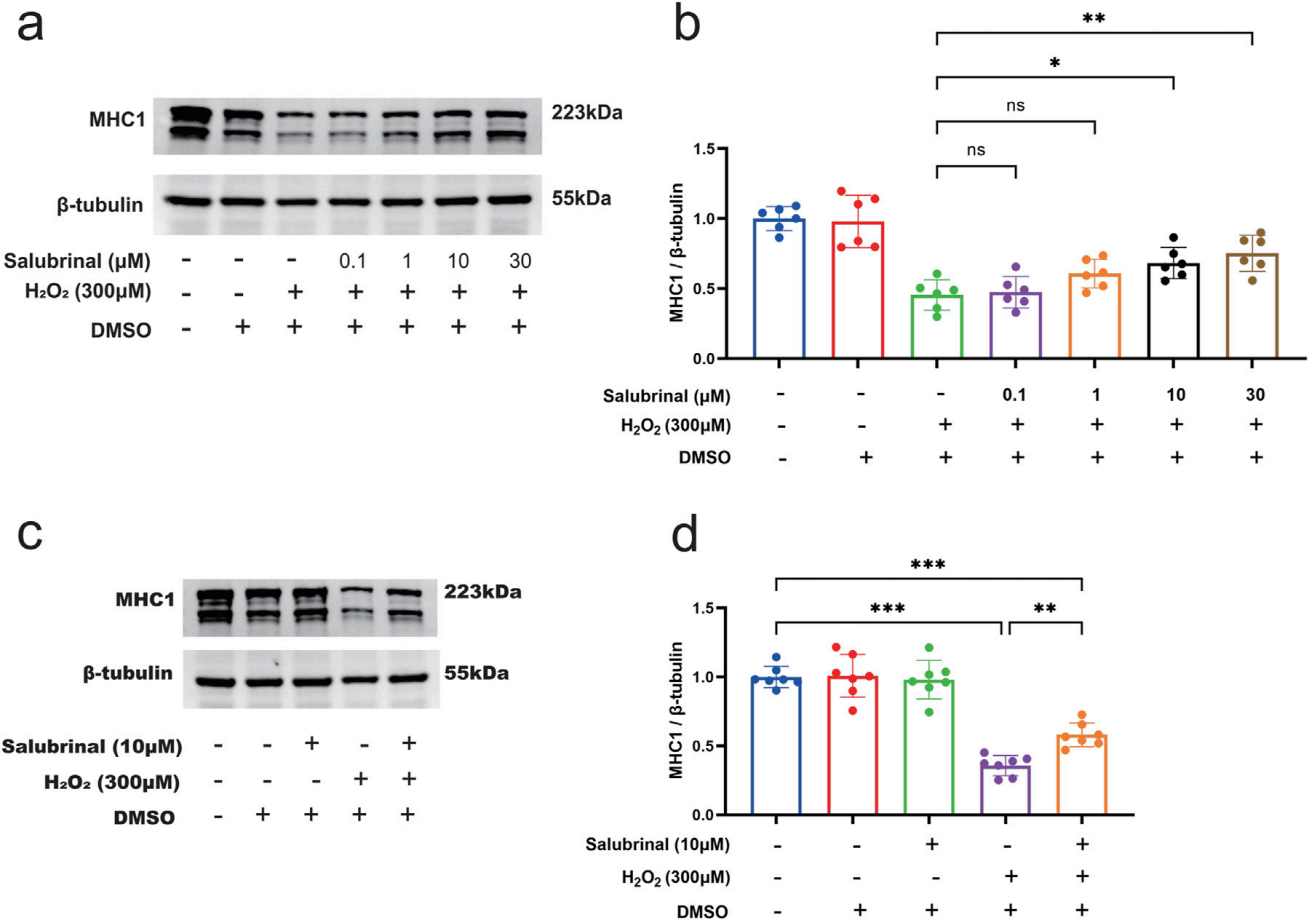
Effect of salubrinal on H_2_O_2_-induced myotube damage. **(a)** Western blot detection of different concentrations (0, 0.1, 1, 10, and 30 μmol/L) of salubrinal on the expression of MHC1 in C2C12 myotube cells treated with H_2_O_2_ for 48 h. β-tubulin was used as the loading control. **(b)** Quantitative analysis of the effect of different concentrations of salubrinal on the expression level of MHC1 in C2C12 myotube cells treated with H_2_O_2_ for 48 h. Data are presented as mean ± standard deviation. ns, no significance, *P < 0.05, **P < 0.01, ***P < 0.001, n = 6 independent experiments. **(c)** The effect of 10 μmol/L salubrinal on the expression level of MHC1 in C2C12 myotube cells treated with 300 μmol/L H_2_O_2_ for 48 h by Western blot. β-tubulin was used as the loading control. **(d)** Quantitative analysis of the effect of 10 μmol/L salubrinal on the expression level of MHC1 in C2C12 myotube cells treated with 300 μmol/L H_2_O_2_ for 48 h. Data are presented as mean ± standard deviation. ns, no significance, *P < 0.05, **P < 0.01,***P < 0.001, n = 5 independent experiments.

In order to study the impact of salubrinal on muscle injury induced by H_2_O_2_, we used different concentrations of salubrinal (0.1∼30 μmol/L) to pre-stimulate the cells for 1 h, and then gave 300 μmol/L H_2_O_2_ to intervene C2C12 myotubular cells for 48 h. The results were that salubrinal could present concentration (0.1∼30 μmol/L) dependent inhibition of H_2_O_2_ (300 μmol/L) induced a decrease in MHC1 protein expression in myotubular cells; when the concentration of salubrinal reached 10 μmol/L and above, the level of MHC1 protein expression in myotubular cells in the H_2_O_2_ + salubrinal group was significantly higher compared with the H_2_O_2_ group (*P* < 0.05) (Figures 5a,b). Therefore, subsequent treatment with 10 μmol/L salubrinal to explore the effect of salubrinal.

**FIGURE 6:**
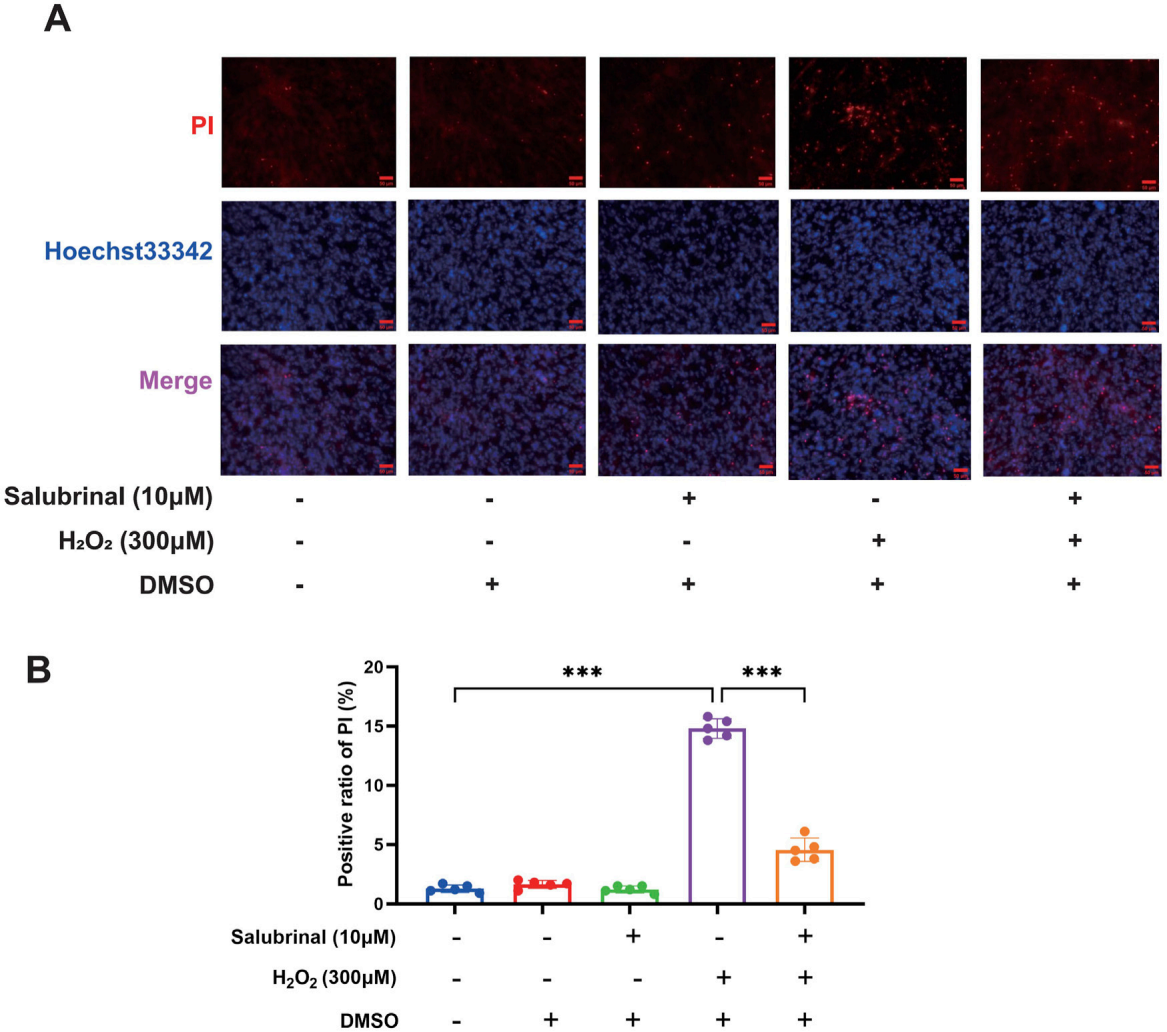
The effect of salubrinal on H_2_O_2_-induced myotube apoptosis (PI method). **(A)** Hoechst33342/PI fluorescent double staining was used to detect the effect of 10 μmol/L salubrinal on C2C12 myotubes treated with 300 μmol/L H_2_O_2_ for 48 h. Scale bar = 50 μm (magnification ×200). **(B)** Statistical results of the effect of 10 μmol/L salubrinal on the PI positive rate of C2C12 myotubes treated with 300 μmol/L H_2_O_2_ for 48 h. Data are presented as mean ± standard deviation. ns, no significance, *P < 0.05, **P < 0.01, ***P < 0.001, n = 5 independent experiments.

**FIGURE 7:**
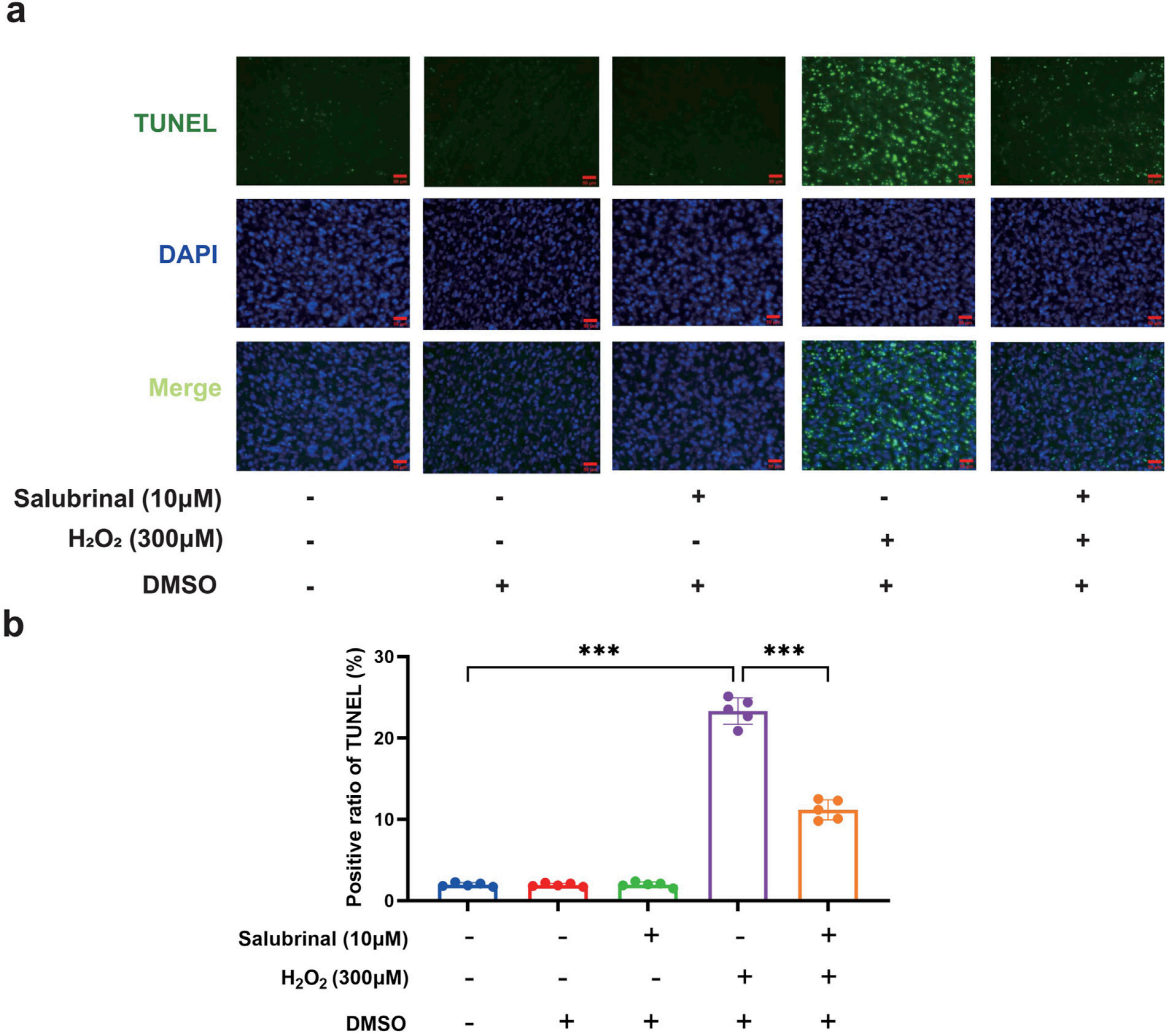
The effect of salubrinal on H_2_O_2_-induced myotube apoptosis (TUNEL method). **(a)** TUNEL staining to detect the effects of 10 μmol/L salubrinal on C2C12 myotubes treated with 300 μmol/L H_2_O_2_ for 48 h. Scale bar = 50 μm (magnification ×200). (b) Statistical results of the effect of 10 μmol/L salubrinal on the positive rate of TUNEL in C2C12 myotubes treated with 300 μmol/L H_2_O_2_ for 48 h. Data are presented as mean ± standard deviation. ns, no significance, *P < 0.05, **P < 0.01, ***P < 0.001, n = 5 independent experiments.

Morphological observation showed that compared with the blank control group, a significant increase in the number of shrunken and curled-up dead cells in the H_2_O_2_ group, and a decrease in the shrunken and curled-up dead myotubes was observed in the H_2_O_2_ + Salubrinal group compared to the H_2_O_2_ group (Supplementary Figure S1); cellular immunofluorescence observation showed that myotubes became slimmer in the H_2_O_2_ group compared to the blank control group, and this alteration was alleviated in the H_2_O_2_ + salubrinal group alleviated this change (Supplementary Figure S2); Western blot analysis revealed a significant reduction in MHC1 expression in the H_2_O_2_ group compared to the control (*P* < 0.05). Conversely, MHC1 levels were significantly elevated in the H_2_O_2_ + Salubrinal group relative to the H_2_O_2_ group (*P* < 0.05) (Figures 5c,d). The results of Hoechst33342/PI fluorescence double staining and TUNEL staining assay showed that compared with the blank control group, the PI positivity rate was significantly higher in the H_2_O_2_ group (*P* < 0.05), and compared with the H_2_O_2_ group, a significant decrease in the PI positivity rate was seen in the H_2_O_2_ + Salubrinal group (*P* < 0.05) (Figure 6); TUNEL positivity was significantly higher in the H_2_O_2_ group compared to the blank control group (*P* < 0.05), and TUNEL positivity was significantly lower in the H_2_O_2_ + Salubrinal group compared to the H_2_O_2_ group (*P* < 0.05) (Figure 7).

### 3.5 Effect of Salubrinal on H_2_O_2_-induced eIF2α/ATF4 pathway in myotubular cells

**FIGURE 8:**
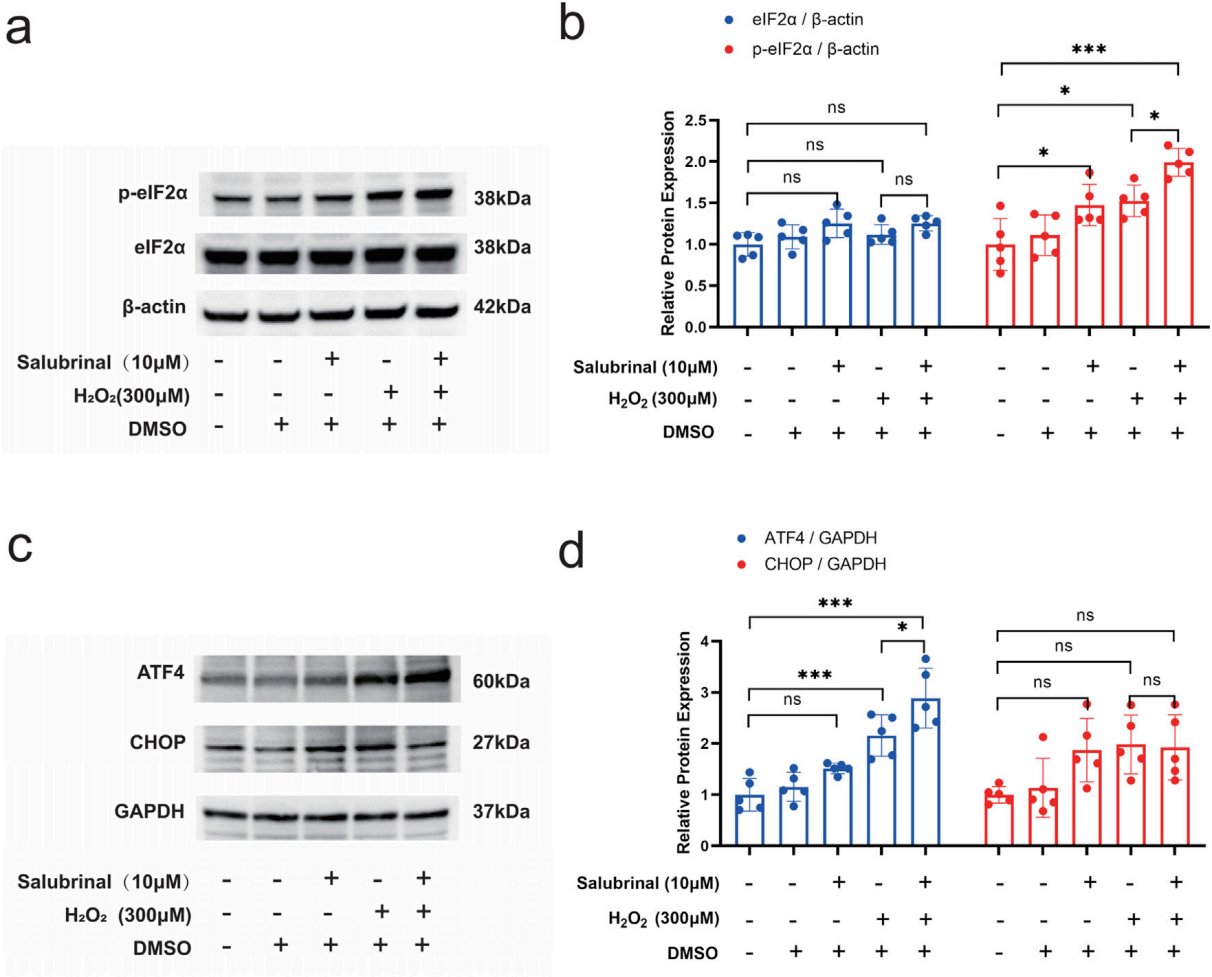
Effect of salubrinal on the H_2_O_2_-induced eIF2α/ATF4 pathway in myotubular cells. **(a)** The effect of 10 μmol/L salubrinal on the expression level of p-eIF2α and eIF2α in C2C12 myotube cells treated with 300 μmol/L H_2_O_2_ for 24 h by Western blot. β-actin was used as the loading control. **(b)** Quantitative analysis of the effect of 10 μmol/L salubrinal on the expression level of p-eIF2α and eIF2α in C2C12 myotube cells for 24 h after 300 μmol/L H_2_O_2_ treatment. **(c)** Treated with 300 μmol/L H_2_O_2_ for 24 h by Western blot. GAPDH was used as the loading control. **(d)** Quantitative analysis of the effect of 10 μmol/L salubrinal on the expression level of ATF4 and CHOP in C2C12 myotube cells for 24 h after 300 μmol/L H_2_O_2_ treatment. Data are presented as mean ± standard deviation. ns, no significance, *P < 0.05, **P < 0.01, ***P < 0.001, n = 5 independent experiments.

Salubrinal was discovered to have a protective impact on musclei njury induced by H_2_O_2_. While it is known as a specific inhibitor of eIF2α dephosphorylation (Boyce et al., 2005), it remains uncertain if the protective effect is due to its influence on the eIF2α-related signaling pathway. Western blot assay showed that p-eIF2α and ATF4 protein expression levels were significantly increased in the H_2_O_2_ group, and the protein expression levels of p-eIF2α and ATF4 were further significantly increased in the H_2_O_2_ + Salubrinal group compared with the H_2_O_2_ group (*P* < 0.05). At the same time, the protein expression levels of CHOP tended to decrease but did not reach a statistically significant difference (Figure 8).

## 4 Discussion

Previous studies have shown a significant increase in Atrogin-1 and MuRF-1 levels in the gastrocnemius muscle of aged mice compared to younger groups, indicating a strong correlation with muscle atrophy during aging. (Supplementary Figure S3). In this study, we examined the expression of muscle ERS-related proteins GRP78 and CHOP, from the samples of our preveous study, and presents pivotal findings on the protective effects of salubrinal against H_2_O_2_-induced muscle wasting in cell experiments. Firstly, significant muscle atrophy and ERS were found in the gastrocnemius muscle of aged mice. Secondly, it was found that salubrinal significantly ameliorates H_2_O_2_-induced damage in myotubes. The study is significant in showing that salubrinal’s protection is linked to the eIF2α/ATF4 signaling pathway, supported by the rise in p-eIF2α and ATF4 levels when salubrinal is present. Additionally, salubrinal was found to reduce muscle cell apoptosis, further underscoring its therapeutic potential in sarcopenia and muscle wasting conditions.

Muscular fibrosis is known to be induced by advanced age. The increase in Atrogin-1 and MuRF-1 expression in aged mice highlights the role of ubiquitin-proteasome pathways in muscle atrophy, consistent with findings from Bodine et al. (2001) and Schiaffino et al. (2013), who emphasized the importance of these pathways in muscle protein degradation. The elevated levels of GRP78 and CHOP suggest an enhanced ERS response in aged muscle, aligning with the work of Chalil et al. (2015) and Deldicque (2013), which pointed out the critical role of ERS in muscle aging and atrophy.

It has been shown that ERS-activated UPR protects stressed cells by reducing protein synthesis and thus mitigating ERS, on the one hand, restoring endoplasmic reticulum homeostasis by decreasing the burden on the endoplasmic reticulum through protein folding to a certain extent; on the other hand, since the former regulatory capacity is limited, it initiates apoptosis under conditions of sustained stress and unrepairable cellular damage to eliminate these stressed cells. UPR is mainly mediated by three endoplasmic reticulum transmembrane receptors: IRE1, PERK and ATF6 (Ramirez et al., 2019). They are transmembrane proteins located in the endoplasmic reticulum membrane. Under normal conditions, they are bound to the chaperone molecule GRP78 and remain in an inactive state. When ERS occurs, unfolded or misfolded proteins recruit GRP78, which results in the dissociation of GRP78 from IRE1, ATF6, and PERK, and after dissociation IRE1, ATF6, and PERK are activated and further activate downstream UPR signaling (Frakes and Dillin, 2017).

Studies have shown that the three main signaling pathways of the UPR (PERK-eIF2α, IRE1α-XBP-1 and ATF6 (Kato and Nishitoh, 2015) tend to be activated in most stressful situations, thus making it more difficult to study the role of the UPR in pathophysiological processes related to skeletal muscle (Pierre et al., 2014). Several recent studies have focused on the PERK-eIF2α signaling pathway. Studies have shown that mice overexpressing PERK, compared to wild-type mice, lose weight and have decreased muscle strength and muscle mass in adulthood, and that sustained activation of the PERK pathway inhibits protein synthesis by promoting phosphorylation of eIF2α leading to a decrease in muscle mass, but enhances expression of antioxidant genes through specific promotion of ATF4 expression leading to healthier skeletal muscle (Miyake et al., 2016). However, mice with specific knockout of PERK also showed decreased muscle mass and muscle atrophy, although knockout of PERK caused a decrease in the ratio of p-eIF2α/eIF2α and an increase in protein synthesis, it also induced the activation of the ubiquitin proteasome pathway, autophagy pathway, and ultimately caused skeletal muscle atrophy (Gallot et al., 2019). Given these seemingly contradictory findings, the role of the PERK-eIF2α pathway in muscle needs to be explored in further studies.

Research has demonstrated that when skeletal muscle cells are exposed to H_2_O_2_, it leads to harm at the cellular level, such as apoptosis (Yin et al., 2015), oxidative stress (Kang et al., 2015), endoplasmic reticulum stress (Pierre et al., 2014), autophagy (Kaur et al., 2019), and mitochondrial dysfunction (Fan et al., 2010). Our study aimed to target endoplasmic reticulum stress to explore the molecular mechanisms of Sarcopenia, so we chose H_2_O_2_ stimulation of C2C12 myotubular cells as a model of muscle injury. Our study showed that H_2_O_2_ could induce myotube injury in a concentration- and time-dependent manner.

Salubrinal, a synthetic endoplasmic reticulum stress inhibitor, is a small molecule compound targeting the eIF2α pathway that selectively binds to the PP1/GADD34 complex, inhibits PP1 activity, preventing the dephosphorylation of eIF2α, and helping to increase the phosphorylation of eIF2α, ultimately reducing apoptosis caused by endoplasmic reticulum stress. Meanwhile, salubrinal is characterized by high safety and selectivity; low cytotoxicity, without toxic cellular effects at a maximum concentration of 100 μmol/L; and as a selective protein phosphatase 1 (PP1)/growth arrest and DNA damage-inducible gene 34 (GADD34) inhibitor, it can only selectively inhibit only a few PP1 complexes in the body and has no effect on dozens of other PP1 complexes (Boyce et al., 2005). Further studies have shown that salubrinal has therapeutic effects on tumors (Kardos et al., 2020; Alsterda et al., 2021), arthritis (Wang et al., 2021), and paraquat poisoning (Wang et al., 2018), but very few studies have been reported on its application in skeletal muscle cytoprotection. In our study, we found that salubrinal’s effect on inhibiting the H_2_O_2_-induced decrease in MHC1 expression and reducing apoptosis through concentration-dependent mechanisms sheds light on its potential as a therapeutic agent. This finding is in harmony with the research by Matsuoka and Komoike (2015), who demonstrated salubrinal’s efficacy in protecting cells from ERS-induced apoptosis.

Several studies have demonstrated that salubrinal effectively impacts oxidative stress. For instance, salubrinal reduces cell apoptosis caused by oxidative stress by inhibiting eIF2α dephosphorylation, which promotes cell survival (Boyce et al., 2005). This is especially relevant in models of oxidative stress, like H_2_O_2_-induced cell damage, where reactive oxygen species disrupt cellular homeostasis. Salubrinal maintains higher levels of phosphorylated eIF2α, helping to reduce oxidative burden and preventing protein misfolding under stress. Our study found that salubrinal alleviated the negative effects of H_2_O_2_-induced oxidative stress in muscle cells, consistent with its protective effects against ROS-induced damage in other cell types (Goswami et al., 2016; Chen et al., 2021). Therefore, it is evident that salubrinal not only protects against endoplasmic reticulum stress but also plays a role in mitigating oxidative stress through its action on the eIF2α/ATF4 signaling pathway.

The involvement of the eIF2α/ATF4 pathway in salubrinal’s protective effects against muscle wasting adds a new understanding of our knowledge in muscle atrophy’s molecular mechanism. It has been shown that phosphorylated eIF2α rapidly and efficiently inhibits translation initiation, thereby effectively blocking most protein synthesis (Harding et al., 1999); the result is a reduction in the entry of newly synthesized proteins into the endoplasmic reticulum, which in turn reduces overloading of the need for folding of newly synthesized proteins. However, upon eIF2α phosphorylation, not all mRNA translation is terminated, and the translation of the transcription factor ATF4 is selectively upregulated; the specific mechanism is that the open reading frame upstream of its mRNA 5′-untranslated sequences, which is formed in a spatial conformation under non-stressful conditions prevents the translation of ATF4’s open reading frame, and the phosphorylation of eIF2α causes the ribosome to phosphorylation of eIF2α causes the ribosome to ignore the initiation of its upstream open reading frame during scanning and directly start the translation of the ATF4 open reading frame. ATF4 has both pro-apoptotic and pro-survival roles, and induces the expression of proteins related to cellular metabolism, redox homeostasis, apoptosis, cellular autophagy, etc., which depend on the extent of ERS and the intracellular environment (Blais et al., 2004). ATF4 regulates methylenetetrahydrofolate dehydrogenase 2 (MTHFD2), glucose-6-phosphate dehydrogenase (G6PD), phosphoglycerate dehydrogenase (PHGDH), and phosphoserine aminotransferase 1 (PSAT1) series at the transcriptional level, which are involved in regulating the expression of enzymes related to nicotinamide adenine dinucleotide phosphate (NADPH) production and play an antioxidant role, thus affecting the endoplasmic reticulum homeostasis and cell apoptosis (Wang et al., 2022). ATF4 can also regulate CHOP at the transcriptional level, which is a pro-apoptotic transcription factor that regulates the transcription of apoptosis-related genes and whose sustained activation induces apoptosis (Ikebe et al., 2020).

ATF4 is involved in the endoplasmic reticulum stress response, protein folding, and calcium signaling. Several studies indicate that ATF4 regulates calcium homeostasis and pathways related to cellular survival and stress responses. For example, Xiang et al. reported that the eIF2α-ATF4 pathway is activated by changes in the calcium environment and is involved in bone regeneration, indicating a close relationship between calcium signaling and this pathway (Xiang et al., 2021). Moreover, Hinton et al. demonstrated that ATF4-dependent increases in mitochondrial-endoplasmic reticulum tethering after OPA1 deletion highlight ATF4’s potential role in calcium regulation (Hinton et al., 2024). Although our study did not directly examine the effects of H_2_O_2_ + Salubrinal treatment on calcium signaling, these findings suggest that salubrinal may regulate calcium signaling by enhancing p-eIF2α and ATF4 expression. This aspect warrants further investigation in future studies.

Our research revealed that salubrinal improved H_2_O_2_-induced muscle damage and enhanced ATF4 expression in muscle cells. Additionally, the levels of CHOP protein expression showed a slight decrease without reaching statistical significance. This indicates that salubrinal’s muscle protection benefits may be attributed to the promotion of survival-related genes through ATF4.

Besides its involvement in the eIF2α/ATF4 signaling pathway, ATF4 also regulates mitochondrial function, shape, and content in skeletal muscle cells. Recent studies, including Memme et al. (2023), show that ATF4 affects mitochondrial dynamics during muscle cell differentiation, which directly influences mitochondrial content and shape. Additionally, ATF4 is crucial in the mitochondrial stress response, as noted by Quirós et al. (2017), where it modulates different mitochondrial proteins in response to cellular stress. Furthermore, Hinton et al. (2024) found that ATF4-dependent tethering between mitochondria and the endoplasmic reticulum is vital for maintaining cellular homeostasis during stress. These findings indicate that salubrinal’s protective effects against oxidative stress and muscle cell apoptosis may involve ATF4-mediated regulation of mitochondrial function.

Our study observed an increase in CHOP expression following H_2_O_2_-induced stress, consistent with previous findings linking elevated CHOP levels to muscle atrophy and cell death (Zheng et al., 2023; Michel et al., 2025). However, the reduction in CHOP expression following salubrinal treatment did not reach statistical significance, indicating a more complex regulation of CHOP under our experimental conditions. Our results indicated the necessity for future studies to thoroughly investigate CHOP’s regulatory network and its precise role in muscle injury and recovery.

ATF4 exerts dual roles in skeletal muscle, mediating either adaptive responses or apoptosis depending on stress intensity and duration. In our study, ATF4 expression correlated with protective effects of salubrinal, suggesting a context where ATF4 activation favors cell survival. Previous research has demonstrated that salubrinal’s modulation of ATF4 can ameliorate degenerative changes in lung and neurons (Wang et al., 2018; Mizugaki et al., 2022), supporting its potential translational value.

**FIGURE 9:**
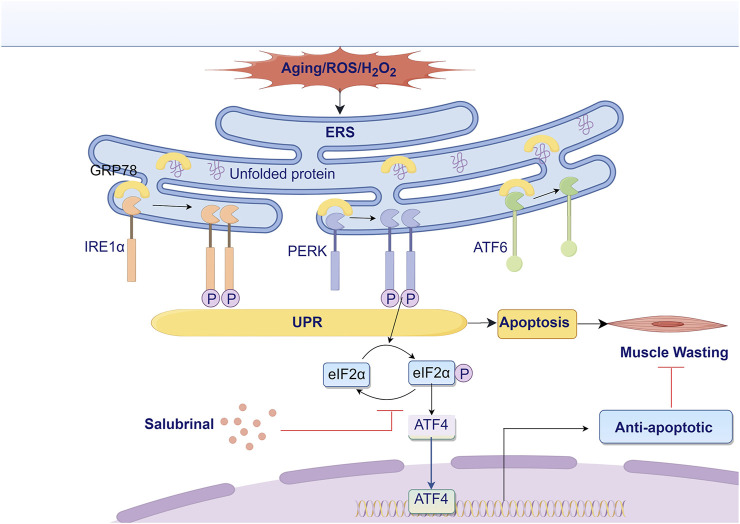
Research hypothesis diagram. ATF4, activating transcription factor 4; ATF6, activating transcription factor 6; eIF2α, eukaryotic translation initiation factor 2α; ERS, endoplasmic reticulum stress; GRP78, glucose-regulated protein 78; IRE1α, inositol-requiring enzyme 1α; PERK, protein kinase RNA-like ER kinase; ROS, reactive oxygen species; UPR, unfolded protein response.

Overall, our tests showed that salubrinal, a compound that blocks endoplasmic reticulum stress, reduces damage to myotube cells caused by H_2_O_2_. This effect is likely due to how it affects the eIF2α/ATF4 pathway, leading to anti-apoptotic outcomes, as illustrated in Figure 9. Our initial studies investigated the possible use of salubrinal as a treatment for Sarcopenia.

Although the study shows strong support for salubrinal’s protective effects, several limitations need to be addressed in future research. We acknowledge the limitation of our study in not directly examining the roles of additional markers such as ATF6, XBP1, pPERK, p62, and LC3B, which could provide deeper insights into autophagy and unfolded protein response mechanisms. Future research should focus on these additional pathways to fully elucidate the complex interaction between ER stress, oxidative stress, and autophagy in muscle wasting. Furthermore, the downstream effects of activating the eIF2α/ATF4 pathway, particularly the roles of CHOP and other stress-responsive genes, require further investigation to fully elucidate salubrinal’s mechanism of action. Future studies should also investigate the long-term effects of salubrinal treatment on muscle function and atrophy in aging and disease contexts.

Despite supporting evidence from previous studies (Lee et al., 2021; Mizugaki et al., 2022) for the use of 300 µM H_2_O_2_ for oxidative stress induction in muscle cells, we recognize the lack of ROS or viability assay data as a limitation in our model validation. Future studies will include ROS quantification and cell viability testing to provide direct evidence of oxidative injury.

While we included TUNEL staining to evaluate apoptosis, we acknowledge that assessing specific injury markers and oxidative stress indicators would provide deeper insights into the mechanisms of H_2_O_2_-induced muscle injury. Previous studies have confirmed the elevation of these markers in H_2_O_2_-induced muscle cell injury models, supporting the role of oxidative stress in mediating cellular damage (Pierre et al., 2014; Kang et al., 2015; Yin et al., 2015). Due to resource limitations, we were unable to perform these assays; however, we consider this an important limitation. In future studies, we will incorporate these indicators to more precisely characterize the cellular injury mechanisms and better evaluate the protective effects of salubrinal in oxidative stress-related myopathy.

Although we observed concurrent activation of ER stress and oxidative stress following H_2_O_2_ treatment, we did not include experiments involving antioxidant intervention to delineate their causal relationship. Prior research has shown that mitigating oxidative stress can suppress ER stress pathways in multiple cell types (Wang et al., 2016; Esmaeili et al., 2022). In future studies, we aim to investigate whether pharmacological inhibition of oxidative stress using agents such as N-acetylcysteine or mitoTEMPO can modulate ER stress and apoptosis in our model. Such work would further clarify the mechanistic role of salubrinal in the context of oxidative and ER stress cross-talk.

Salubrinal-treated cells showed no overt signs of cytotoxicity, consistent with previous reports suggesting its relative safety at the concentration used (Boyce et al., 2005; Matsuoka and Komoike, 2015). However, further dose-response studies will be conducted to confirm its safety profile.

While our study demonstrates the regulatory effects of salubrinal on the eIF2α/ATF4 signaling pathway *in vitro*, we acknowledge the absence of *in vivo* validation as a limitation. Previous research has confirmed the relevance of this pathway in animal models of muscle atrophy (Adams et al., 2017; Montori-Grau et al., 2022; Zheng et al., 2023). In future work, we aim to establish *in vivo* models to further verify the protective role of salubrinal in skeletal muscle and enhance the translational value of our findings.

## 5 Conclusion

In conclusion, we found an increase in gastrocnemius ERS levels in elderly mice, confirming that salubrinal can improve H_2_O_2_-induced myotube cell injury, and the mechanism may be related to salubrinal’s regulation of the eIF2α/ATF4 pathway. Further study of salubrinal’s muscle-protecting role in animals is needed in the future.

## Data Availability

The raw data supporting the conclusions of this article will be made available by the authors, without undue reservation.

## Supplementary material

SUPPLEMENTARY FIGURE S1 The effect of salubrinal on morphological changes indued by H_2_O_2_ in myotubes.

SUPPLEMENTARY FIGURE S2 The effect of salubrinal on the expression and localization of MHC1 indicated by immunofluorescence in myotube cells induced by H_2_O_2_.

SUPPLEMENTARY FIGURE S3 Changes in the expression levels of Atrogin-1 and MuRF-1 in the gastrocnemius muscle of mice during aging.
