# Supplementary figures and images for: Protective effects of Salubrinal against H2O2-induced muscle wasting via eIF2α/ATF4 signaling pathway
Source: Front Pharmacol. 2025 Jun 27;16:1607606. doi: 10.3389/fphar.2025.1607606 (PMC12245859; doi:10.3389/fphar.2025.1607606)

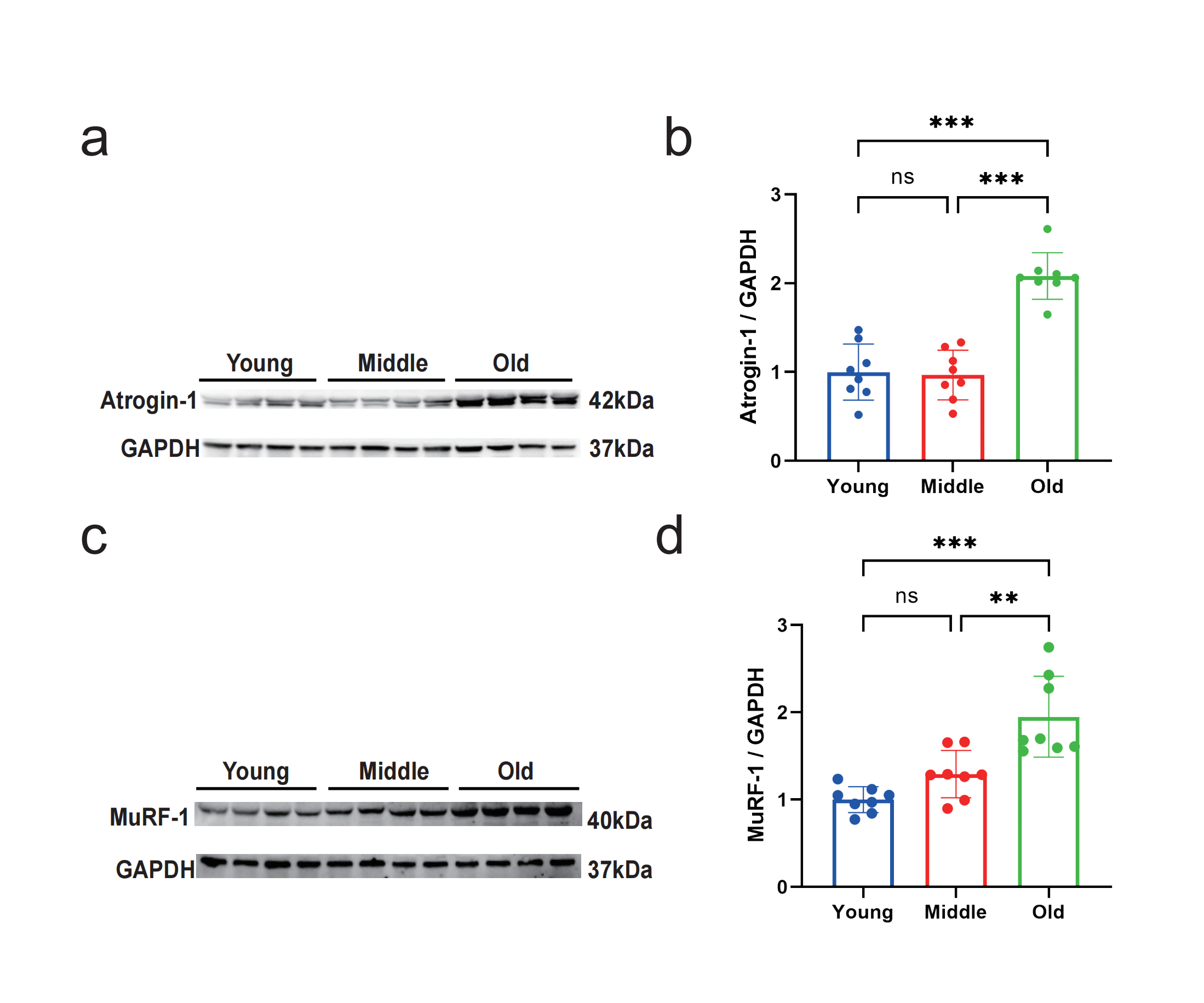

Supplement: Supplementary file 1 [file Image3.tif]

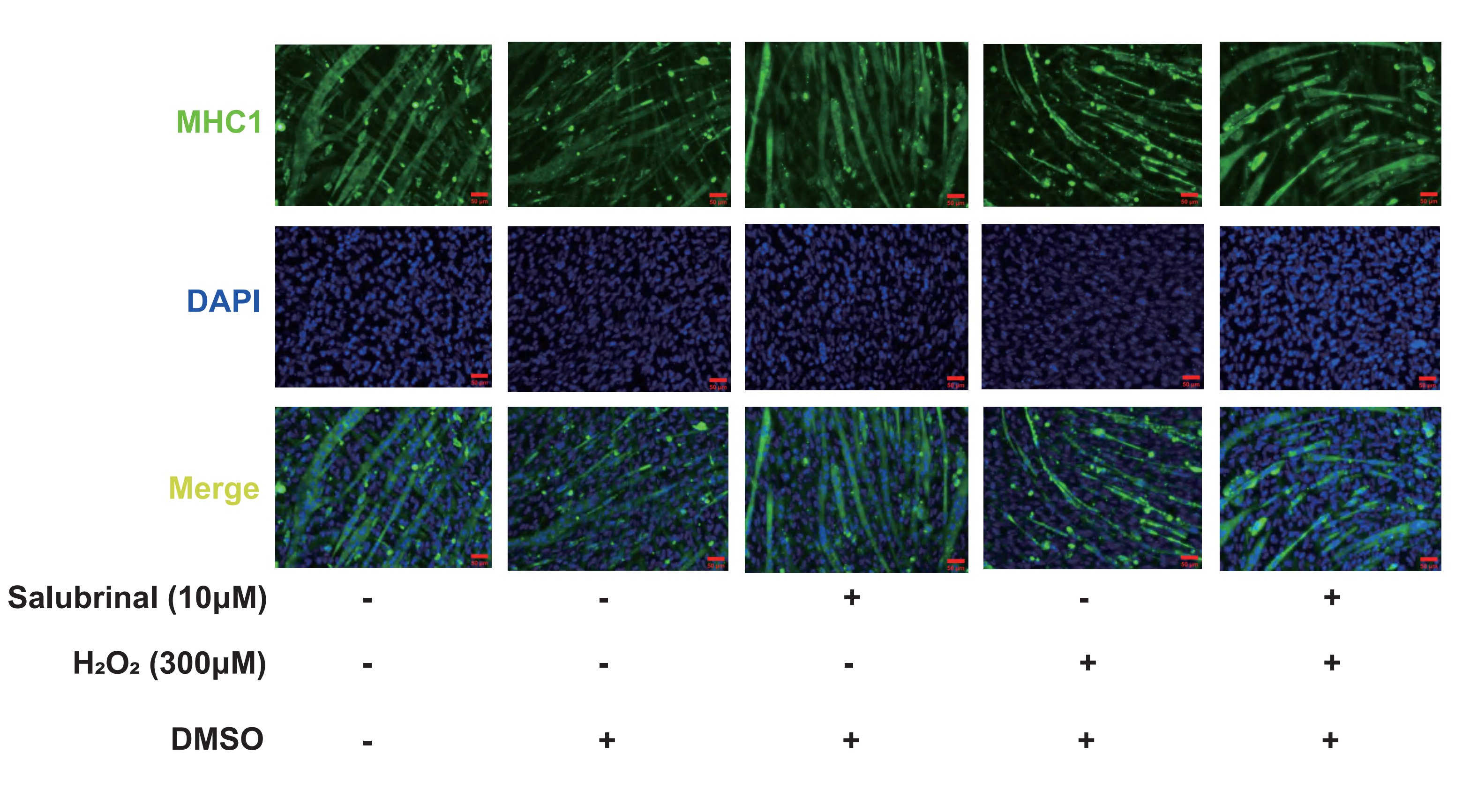

Supplement: Supplementary file 2 [file Image2.tif]

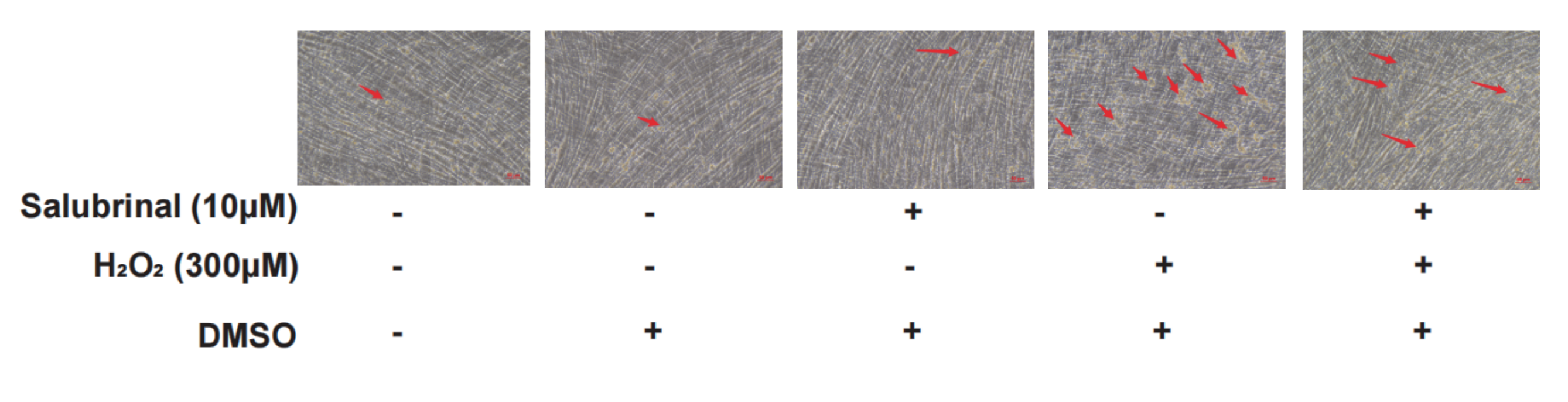

Supplement: Supplementary file 3 [file Image1.tif]
